# Bag-breakup fragmentation as the dominant mechanism of sea-spray production in high winds

**DOI:** 10.1038/s41598-017-01673-9

**Published:** 2017-05-09

**Authors:** Yu. Troitskaya, A. Kandaurov, O. Ermakova, D. Kozlov, D. Sergeev, S. Zilitinkevich

**Affiliations:** 1 Institute of Applied Physics, Nizhny Novgorod, Russia; 2 Division of Atmospheric Sciences, University of Helsinki, Helsinki, Finland; 3 Finnish Meteorological Institute, Helsinki, Finland

## Abstract

Showing the record strengths and growth-rates, recent hurricanes have highlighted needs for improving forecasts of tropical cyclone intensities most sensitive to models of the air-sea interaction. Especially challenging is the nature of sea-spray supposed to strongly affecting the momentum- and energy- air-sea fluxes at strong winds. Even the spray-generation mechanisms in extreme winds remained undetermined. Basing on high-speed video here we identify it as the *bag-breakup* mode of fragmentation of liquid in gaseous flows known in a different context. This regime is characterized by inflating and consequent bursting of the short-lived objects, *bags*, comprising sail-like water films surrounded by massive liquid rims then fragmented to giant droplets with sizes exceeding 500 micrometers. From first principles of statistical physics we develop statistical description of these phenomena and show that at extreme winds the bag-breakup is the dominant spray-production mechanism. These findings provide a new basis for understanding and modeling of the air-sea exchange processes at extreme winds.

## Introduction

The sea spray, a typical feature of the marine atmospheric boundary layer, is one of the most uncertain factors among those controlling hurricanes and severe storms^1–6^. Empirical estimates of the amount and sizes of droplets injected into the atmosphere from the ocean surface are uncertaint up to six orders of magnitude^7^ due to enormous difficulties in field experiments in extreme winds. The very mechanisms of the spray production are still not fully understood^4, 7^.

To investigate how extremely strong winds tear off spray from wave crests, to classify such events, and to quantify the efficiency of the disclosed mechanisms we performed experiments in high-speed wind-wave flume^8^ employing high-speed video-filming (see details of the experimental setup in Methods and Supplementary Materials, Section A1).

The scaling parameter for the air turbulent boundary layer flow above the water surface is the friction velocity, u_*_, defined via vertical turbulent shear stress: ${F}_{M}={\rho }_{air}{u}_{\ast }^{2}$, where *ρ*_*air*_ is the air density. In our experiments the friction velocity, u_*_, varied between 0.8 and 1.5 m/s. According to known empirical relationship^9^, in the field conditions these values correspond to the wind speeds at the reference height 10 m, *U*_*10*_, between 18.4 (Beaufort number 8)^10^ and 35 m/s (Category 1 hurricane)^11^.

Basing on video-filming done at the rates up to 10000 fps we formulate the following classification of spray-generating mechanisms.

1. Breaking projection (Fig. 1A and video S1)

**Figure 1:**
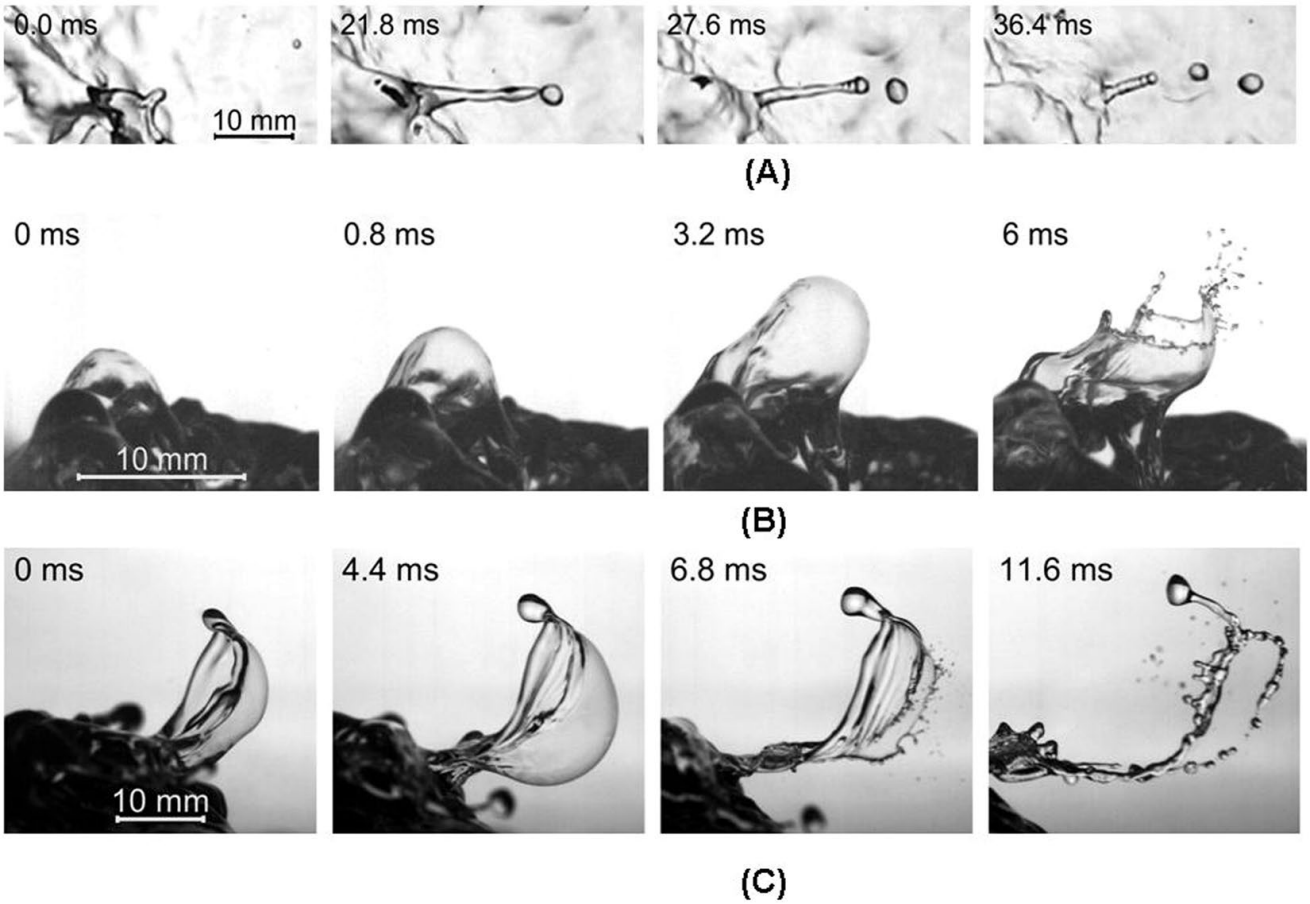
Mechanisms of spray generation(wind blow from left): (**A**) breaking projection (top view); (**B**) rupture of large bubble (side view); (**C**) formation and rupture of bag (side view).
  Small “projections”^12^ develop mainly at crests of breaking waves and break into a few droplets with 1–2 millimetre diameters.
2. Underwater bubble-bursting (Fig. 1B and video S2)
  Underwater bubbles forming at crests of breaking waves burst into droplets when reach the water surface^13^. This mechanism is usually considered as a major one responsible for generation of marine aerosol^13^. Contrastingly, our video-filming has revealed its surprising inefficiency: only about 5% of the observed large underwater bubbles reach water surface and burst to produce droplets.
3. Bag-breakup (Fig. 1C and video S3)

Typical event of this type starts with a small-scale elevation of the water surface, which then develops into a “micro sail”, inflates into a water film bordered by thicker rim, and finally blows up producing hundreds of spray. Processing video-frame consequences allowed us to determine the distribution of bags in radii, *R*, and life-times, τ. Mean values of these parameters, roughly estimated as 〈*R*〉 ~ 10^−2^ m and 〈*τ*〉 ~ 10^−2^ s, generally decrease with increasing wind speed (see Equations (S3–S5) in Supplementary Materials). In engineering fluid dynamics^14^ this phenomenon is known as the bag-breakup mode of liquid fragmentation in gaseous flows. Recently an evidence of the bag-breakup spray generation in laboratory flume was also reported^15^.

**Figure 2:**
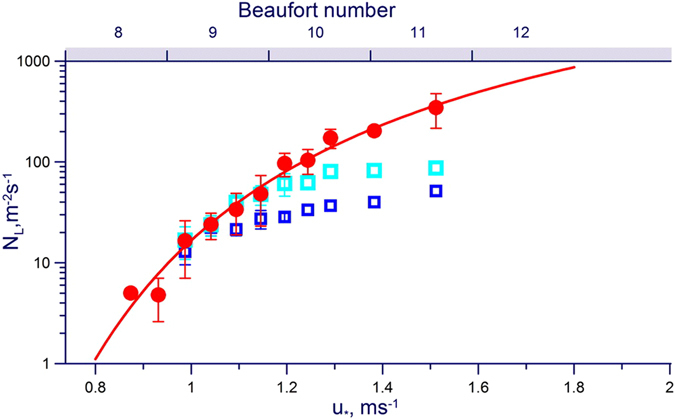
Number of local spray generating phenomena per unit time per unit area versus the friction velocity u_*_ and the Beaufort number: blue squares – bursts of floating bubbles, cyan squares – projections, red circles – bag breakup; red solid curve is given by Equation (1).

Comparative efficiency of the above mechanisms quantified by processing about 2.3 million video-frames (see Supplementary Materials for details of the algorithm) is illustrated in Fig. 2, where each mechanism is characterized by the specific number of events arisen per unit time over unit area as dependent on the friction velocity, u_*_. At u_*_ < 1 m/s the three mechanisms are almost equally efficient, but at u_*_ > 1 m/s the number of bursting bubbles lags essentially behind the numbers of projections and bags. Moreover, breaking projections yield up to 3 droplets per event; so the bag-breakups, yielding about one hundred of droplets per event, become absolutely dominant. Note, that the threshold of the bag-breakup regime in our experiments was u_*_ ≈ 0.9 m/s, which corresponds to *U*_10_ ≈ 20 m/s^9^ that is to the Beaufort Number 8^10^ (see Fig. 2). It is interesting to note, that the Beaufort scale based on the sea’s appearance describes the criterion of the ‘Force 8 wind’ or gale as the beginning of breaking edges of wave-crests into the spindrift: the sea spray being torn by a violent wind. This observation suggests that the spindrift visualizes triggering of a new spray-generation regime with the threshold at the gale force wind.

The specific number, 〈*N*〉, of the bag-breakup events per unit time per unit area can be approximated by the simple expression

1 $$\langle N\rangle ={N}_{0}\frac{{u}_{\ast }^{2}}{{U}_{0}^{2}}\exp (-\frac{{U}_{0}^{2}}{{u}_{\ast }^{2}}),$$

which follows from phenomenological statistical-physics approach using the Gibbs method^16^ (details see in Supplementary Materials, Section A2). The best fit to experimental data illustrated in Fig. 2 gives empirical constants *U*_*0*_ = 2 m/s, *N*_*0*_ = 3.73 · 10^3^ m^−2^ s^−1^.

As seen in Fig. 1C, bags generate spray in two ways:

- Rupturing the film of inflated bag (Fig. 1C, 6.8 ms), which yields film-droplets with average radius ~100 μm.
- Fragmenting the rim, which preserves for a while after the bag’s blow up (Fig. 1C, 11.6 ms), thus yielding rim-droplets with average radius ~1000 μm.

Herewith, the sizes of droplets are prescribed by the sizes of bags. We can expect then, that the bag-breakup sea spray generation function defined as the volume of droplets of radius *r* produced from the unit area of water surface in unit time due to the dominant bag-breakup mechanism has two peaks corresponding to the film- and rim- droplets, respectively.

Our analyses has revealed that the dominant spray-generation mechanism in extreme winds roots in specific instability at the air-water interface known in engineering fluid mechanics as the bag-breakup fragmentation. Coincidence of the activation thresholds enables us to identify the bag-breakup with the spindrift – the signature of the force 8 wind of the Beaufort scale based on the sea’s appearance^10^. In view of this finding, it seems natural to assume that the criterion of the Force 11 wind, namely, “the edges of the wave crests are blown into froth”^10^ is nothing but a manifestation of another yet unknown feature of the air-sea coupling at extreme winds.

## Methods

Our experiments were carried out in the wind-wave flume of Large Thermally Stratified Tank (LTST) of the Institute of Applied Physics Russian Academy of Sciences(IAP RAS). The overall sizes of the facility are 20 meters in length, 4 meters in width and 2 meters in depth. The airflow channel positioned at the top of the LTST with the cross-section of 0.4 × 0.4 m over the water surface had the length of 10 m. The tank was filled with fresh water, with temperature ranging during experiments from 15 to 20 °C. The measured value of the surface tension was σ = (7.0 ± 0.15) · 10^−2^ N/m. The air flow velocities in the facility correspond to the 10 m wind speed in the range between 12 m/s and 40 m/s. The lengths of wind waves in the working section of the facility are between 0.10 and 0.65 m respectively. More details of the facility construction and parameters of air flow and surface waves are described in^8^.

Measurements were carried out in two working sections at fetches 6.5 and 7.5 m. Video filming of the air-water interface was done by the high-speed digital camera NAC Memrecam HX-3 from two different angles: side view – using the vertical matte screen and LED 300 W lights (horizontal shadow method); and top view – using underwater illumination (vertical shadow method).

The side view filming gave us overall views of spray-generating phenomena. The optical axis of the camera lens was located 5 cm above the water surface and directed horizontally. The distance from the camera to the shooting area was 65 cm. A LED spotlight was mounted at the side of the channel section 8 at the distance 50 cm from the wall and the height less than 5 cm from the water surface. A matte screen was placed on the side wall of the channel opposite to the camera. We used the lenses with focal lengths 50 and 85 mm with resolution 55 and 119 μm/px correspondingly. The recording rate was 10000 fps. For wind speeds from 22.2 to 39.5 m/s we obtained less than a second long detailed records of the surface features, while working with the camera records we only selected parts of the records containing the spray generation. Typical images of events leading to the spray generation are shown in Fig. 1(A–C) and movies S1–S3.

To obtain statistical data for the events on the surface leading to spray generation, video-filming was done using the vertical shadow method. Filming was conducted through the transparent top wall of the channel section. Camera was mounted vertically at the distance 207 cm from the water surface. Video-filming was carried out at rates 4500 and 10000 fps with the scales 256 and 124 µm/px, respectively, in the wide range of wind speeds: 22.2–39.5 m/s.

Statistical data for the events on the surface leading to the spray generation was retrieved from video-filming using specially developed software allowed for semi-automatic registering of the events leading to spray generation: breaking projections, bursting underwater bubbles, and “bag-breakup”. The software provided convenient way to browse through recordings at slow speed of frame-by-frame, to find the features of interest and to mark them. Markers were manually added to the image sequences using computer mouse. In total, 69 video-films containing about 33000 frames each were processed to get the statistics.

The scheme of the experimental setup and details of the data processing algorithm are given in Supplementary Materials, Section A1. The theoretical derivation of the statistics of bag-breakup events is presented in Supplementary Materials, Sections A2.

## Electronic supplementary material

Supplementary Materials and Methods

S1

S2

S3

S1
