# Supplementary material for: Bag-breakup fragmentation as the dominant mechanism of sea-spray production in high winds
Source: Sci Rep. 2017 May 9;7:1614. doi: 10.1038/s41598-017-01673-9 (PMC5431630; doi:10.1038/s41598-017-01673-9)
Supplement: Supplementary file 1 — Supplementary Materials and Methods [file 41598_2017_1673_MOESM1_ESM.pdf]

Supplementary Materials and Methods for

Bag-breakup fragmentation as the dominant mechanism of sea-spray production in high winds

Yu. Troitskaya[1]*, A. Kandaurov[1], O. Ermakova[1], D. Kozlov[1], D. Sergeev[1], S. Zilitinkevich[2,3]*.

*Correspondence to: yuliya@hydro.appl.sci-nnov.ru, sergej.zilitinkevich@fmi.fi.

This file includes:

Supplementary Text
Figs. S1 to S4
Captions for Movies S1 to S3
Captions for database S1 to S1

Other Supplementary Materials to this manuscript include:

Movies S1 to S3
Database S1 as a zipped archive including the following data files in ASCII encoding:
n_events.dat - number of bags, projections and underwater bubble-bursting events via friction velocity,
P_size1.dat - the distribution function of "bags" in the radius at the moment of nucleation ($R_1/\langle R_1\rangle$),
P_size2.dat - the distribution function of "bags" in the radius at the moment of rapture ($R_2/\langle R_2\rangle$),
tol.dat - the distribution function of "bags" on their lifetime ($\tau/\langle\tau\rangle$,
speededges.dat - - the distribution function of "bags" on their speeds of edges ($u/\langle u\rangle$,
size1_2_tol(u).dat - average initial and final radii of bags and their average lifetime via wind friction velocity,
f_.dat - initial R_1 and final R_2 radii of "bags".

A1. Experimental setup

The experiments were carried out in the wind-wave flume of Large Thermally Stratified Tank (LTST) of the Institute of Applied Physics Russian Academy of Sciences (IAP RAS). The overall sizes of the facility are 20 meters in length, 4 meters in width and 2 meters in depth. The airflow channel positioned at the top of the LTST with the cross-section of 0.4×0.4 m over the water surface had the length of 10 m. The tank was filled with fresh water, with temperature ranging during experiments from 15 to 20°C. The measured value of the surface tension was $\sigma = (7.0\pm0.15)\cdot10^{-2}$ N/m. The air flow velocities in the facility correspond to the 10m wind speed in the range between 12 m/s and 40 m/s. The lengths of wind waves in the working section of the facility are between 0.10 and 0.65 m respectively. More details of the facility construction and parameters of air flow and surface waves are described in[8].

Measurements were carried out in two working sections spaced 7 and 8 m from the outlet of the fan. To characterize the air flow, we use friction velocity u_* and roughness height z_0 retrieved from the wind speed using the algorithm[8] based on the self-similarity of the velocity profile in the channel flow. Given u_* and z_0 allows calculating the wind velocity at the reference height H_{10} =10 m:

$$U_{10} = \frac{u_*}{\kappa}\ln\frac{H_{10}}{z_0}, \tag{S1}$$

where κ=0.4 is von Karman constant. The velocity range in the centerline airflow was 3-25 m/s, the corresponding friction velocities were 0.8-1.5 m/s and the equivalent 10-m wind speeds varied from 18 to 35 m/s.

Video filming of the air-water interface was done by the high-speed digital camera NAC Memrecam HX-3 from two different angles: side view in section 8 of the channel – using the vertical matte screen and surface LED 300W lights (horizontal shadow method, Fig. S1(A)); and top view in section 7 of the channel – using underwater illumination (vertical shadow method, Fig.S1(B)). The side view filming gave us overall views of spray-generating phenomena. In that case, the camera was placed in a waterproof box attached to the side wall of the channel near section 8 (at 7.5 m fetch; see. Fig. S1(A)). The optical axis of the camera lens was located 5 cm above the water surface and directed horizontally. The distance from the camera to the shooting area was 65 cm. A LED spotlight was mounted at the side of the channel section 8 at the distance 50 cm from the wall and the height less than 5 cm from the water surface. A matte screen was placed on the side wall of the channel opposite to the camera. We used the lenses with focal lengths 50 and 85 mm with resolution 55 and 119 μm/px correspondingly. The recording rate was 10000 fps. For wind speeds from 18 to 35 m/s we obtained less than a second-long detailed records of the surface features, while working with the camera records we only selected parts of the records containing the spray generation. Typical images of events leading to the spray generation are shown in Fig. 1(A-C) and movies S1-S3.

To obtain statistical data for the events on the surface leading to spray generation, video-filming was done using the vertical shadow method (see setup in Fig. S1(B)). Filming was conducted through the transparent top wall of the channel section 7 (6.5 m fetch). Camera was mounted vertically at the distance 207 cm from the water surface. Video-filming was carried out at rates 4500 and 10000 fps with the scales 256 and 124 μm/px, respectively, in the wide range of wind speeds: 18-35 m/s. See typical images in Fig. S2.

Statistical data for the events on the surface leading to the spray generation was retrieved from video-filming using specially developed software allowed for semi-automatic registering of the events leading to spray generation: breaking projections, bursting underwater bubbles, and "bag-breakup". The software provided convenient way to browse through recordings at slow speed of frame-by-frame, to find the features of interest and to mark them. Markers were manually added to the image sequences using computer mouse. In total, 69 video-films containing about 33000 frames each were processed to get the statistics.

The "bag-breakup" was identified as the most efficient mechanism of spray generation and studied in detail. To describe the position, size and evolution of each visible "bag", a set of five markers was used (see fig. S2). Each maker had two coordinates in pixels and frame number. The first two markers in a set were placed at the frame of the bag nucleation – at the edges of the bag. Other three markers were placed at the frame of film puncture: two markers – at the edges of the bag and one marker – at the point of film puncture (or in the middle of the front side of film – in the case of absence of the observed point of puncture).

The software generated the text file containing x and y-axis coordinates of the markers for each record. These files were automatically analyzed to obtain statistical characteristics of "bags" on the records. The following parameters were used:

$R_1=D_1/2$ – half distance between edge markers at the frame of "bag" nucleation (initial "bag" radius);

$R_2=D_2/2$ – half distance between edge markers at the frame of film puncture (final "bag" radius);

τ – lifetime of "bag" from the moment of its nucleation until the moment of the film puncture;

u – the translation velocity of the "bag" edges determined as the distance between midpoints of edge markers on initial an final frames divided by τ.

A2. Statistics of "bag-breakup" events

A2.1. Number of "bags".

To describe the statistics of numbers of the "bag-breakup" events, we use phenomenological approach based on methods of statistical physics, namely the Gibbs method[16], initially introduced in equilibrium statistical mechanics. The central concept of the method is the Gibbs canonical ensemble, or an ensemble of states of thermodynamic system in weak thermal contact with a heat bath, i.e. environment that keeps its temperature unchanged. Then, the probability that the system is in the state characterized by the energy E is determined by the Gibbs or canonical distribution[16]; and the probability that the energy of the system is in the energy-state range $[E, E + dE]$ is equal to:

$$dW = A\exp\left(-E/\beta\right)dE \, . \qquad \text{(S2)}$$

Here the constant A is determined from the normalization condition, that

$$\int_0^\infty A e^{-\frac{E}{\beta}} dE = 1 ,$$

and then $A=1/\beta$.

When considering the statistics of "bags" we assume the canonical ensemble corresponding to the air-ocean interface that can be potentially transformed into "bags" and then atomized into spray. While the "heat bath" is the the marine atmospheric boundary layer, which prescribes the state of the interface Note, that the weakness on the "thermal contact" is provided by the comparatively small aerodynamic resistance of the ocean surface typical even for hurricane conditions. The parameter of the "heat bath", β, can be derived from the commonly recognized Boussinesq analogy[17] between the turbulent velocity fluctuations and the thermal motions of molecules in gas. For the case of molecular motions, β is proportional to the temperature of the heat bath[16] that is to the average kinetic energy of molecules. In the problem under consideration, the analog to the temperature of gas is the kinetic energy density of turbulent fluctuations. In marine atmospheric boundary layer (MABL) the latter is scaled by the wind friction velocity, i.e. $\beta = \gamma u_*^2$.

When the energy of the system under consideration, E, exceeds a certain threshold E_0, the bag-breakup regime is activated and the specific number of "bags" arising in per unit time per

unit area of the water surface is a certain function of the energy *N(E)*. While the average specific number of "bags" $\langle N\rangle$ (per unit time per unit area) is equal to the integral of *N(E)dW* over all states with the energy exceeding E_0, i.e.

$$\langle N\rangle = \int_{E_0}^{\infty} \frac{N(E)}{\beta} e^{-\frac{E}{\beta}} dE \qquad \text{(S3)}$$

Expanding *N(E)* in the vicinity of the threshold E_0, gives

$$N(E) = N(E_0) + \alpha(E - E_0) + ...$$

here both E_0 and α are the intrinsic parameters of the "quasi-thermodynamic system" independent on the parameter of the "heat bath" β. Since E_0 is the activation threshold for the "bag-breakup" regime, then for $E=E_0$ the number of "bags" $N(E_0)$=0 and integration in equation (S3) yields

$$\langle N\rangle = \alpha\beta e^{-\frac{E_0}{\beta}}. \qquad \text{(S4)}$$

Introducing two new constants

$U_0 = \sqrt{E_0/\gamma}; N_0 = \alpha E_0$, finally equation (S2) can be presented as follows:

$$\langle N\rangle = N_0 \frac{u_*^2}{U_0^2} \exp\left(-\frac{U_0^2}{u_*^2}\right), \qquad \text{(S5)}$$

where U_0=2 m/s, N_0=3.73·10^3 m^{-2}s^{-1} are determined from experimental data shown in Fig. 1D.

A2.2. Statistical distribution of parameters of the "bags"

Semi-automatic processing of the obtained video allowed us to investigate statistical distribution of the observed geometrical and kinematic parameters of the "bags". Fig. S3(A,B,C) demonstrates the probability density functions of these values, expressed in dimensionless variables normalized by the average values. Distributions of "bags" versus their initial radii R_1 and the radii at the moment of film rupture R_2 are shown in Fig. S3(A). Figs. S3(B,C) shows the distribution of "bags" versus their edge velocities *u* and lifetimes (from the moment of their occurrence until the moment of the film puncture τ).

Fig. S3(A,B,C) shows that distributions of "bags" versus R_1, R_2, *u*, and τ normalized by the mean values keep roughly the same shape for different friction velocities and is well approximated by the Gamma distribution:

$$P_n(x) = \frac{n^n}{(n-1)!} x^{n-1} e^{-nx}.$$

Here, $x = X/\langle X\rangle$ designates any physical variable: R_1, R_2 , *u* or τ normalized by its mean value: $\langle R_1\rangle$, $\langle R_2\rangle$, $\langle u\rangle$, *or* $\langle\tau\rangle$; *n*=8 for R_1 and R_2 ; *n*=13 for *u*; and *n*=3 for τ.

Fig. S4(A-C) show dependencies of the above mean values on the friction velocity u_*. With increasing u_* (strengthening wind) the sizes of "bags" and life-times decreasing are decreasing. In the range of u_* covered by our experiments the obtained dependencies can be approximated by the following power laws:

$$\langle R_1\rangle = 5.9u_*^{-1},$$

$$\langle R_2\rangle = 9.6u_*^{-1},$$

$$\langle\tau\rangle = 7.7u_*^{-2},$$

and velocity of the edges follows the linear dependence

$$\langle u \rangle = 1.96 + 1.21\, u_*$$

where u_* is measured in m/s, $\langle R_1 \rangle$ and $\langle R_2 \rangle$ - in millimeters and $\langle \tau \rangle$ - in milliseconds.

Also it worth noticing very strong correlation between R_1 and R_2 for individual "bags": the correlation coefficient between R_1 and R_2 is 0.97, and the mean value of the ratio $\langle R_2/R_1 \rangle$ is 1.6 (see Fig. S4D). This is indicative of the self-similarity of the form of “bags” over the range of scales.

References to supplement

17. Boussinesq, J. V. Essai sur la theorie des eaux courantes. *Mémoires présentés par Divers savants à l’Acad. des Sci. Inst. Nat. Fr.* **XXIII**, 1–680 (1877)

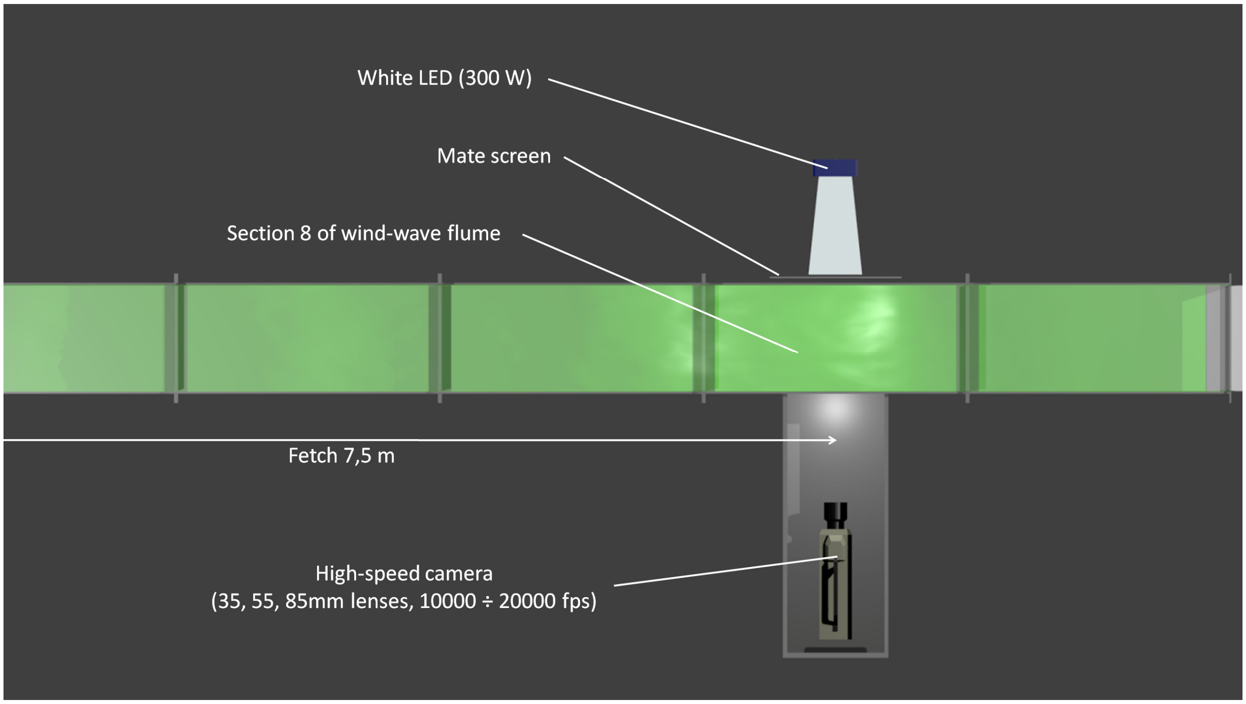

(A)

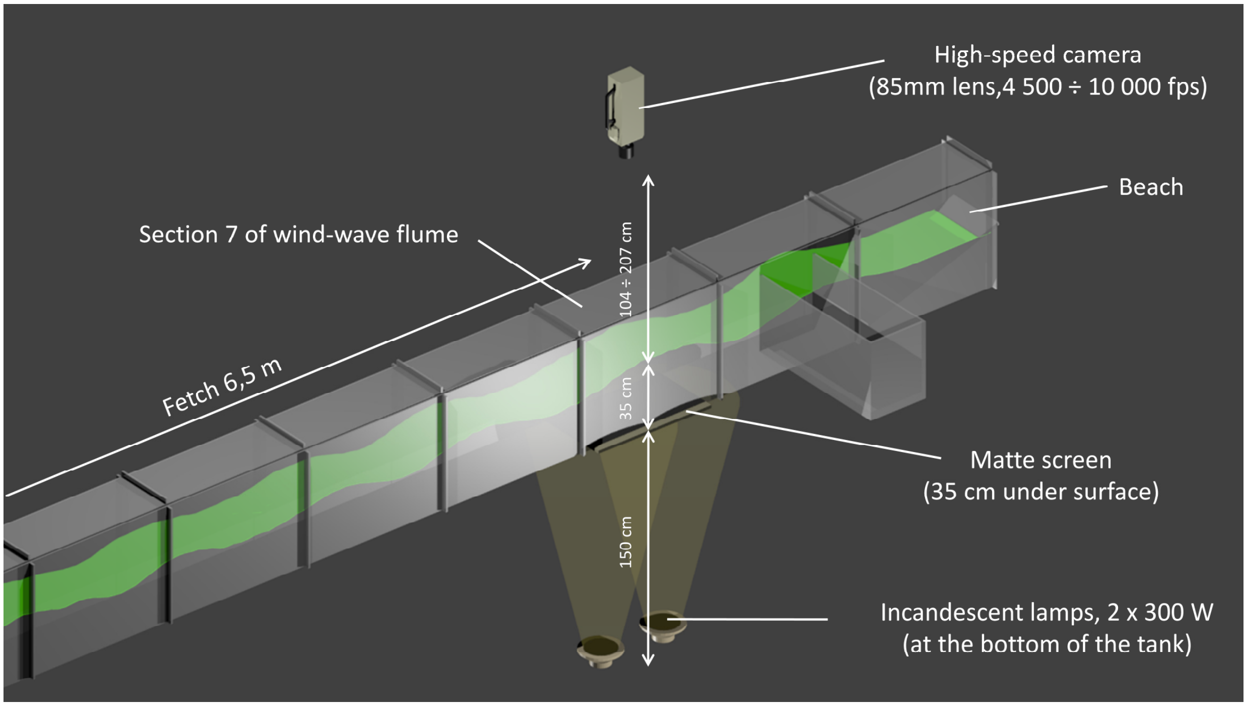

(B)

Fig. S1. Scheme of experiments conducted using the horizontal shadow method (A) and the vertical shadow method (B).

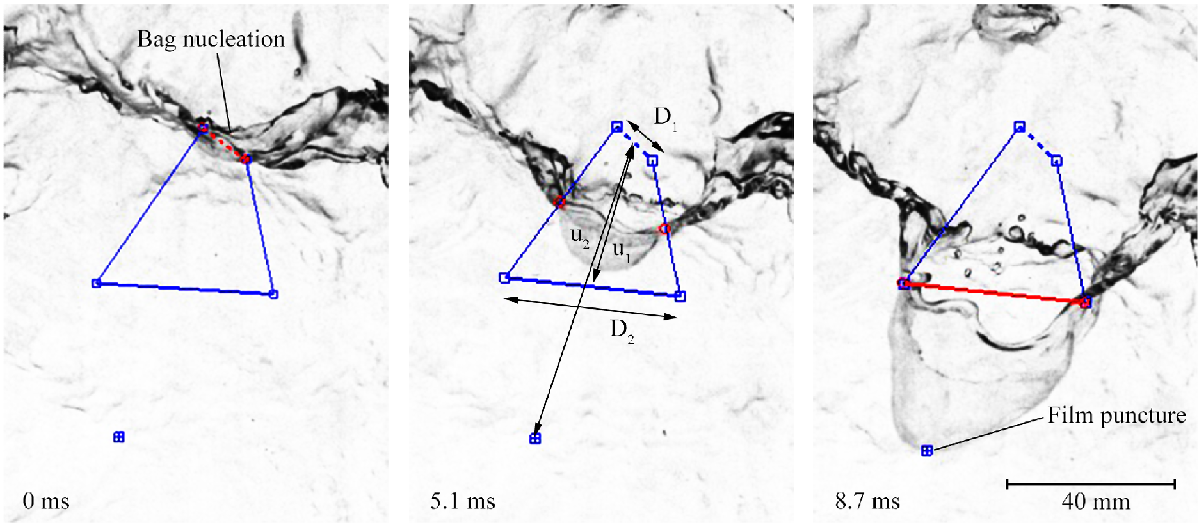

(A) (B) (C)

Fig. S2. Semi-automatic registering of the evolution of bag: (A) initial frame with the bag nucleation, (B) intermediate frame with annotated markers; edge positions are interpolated; (C) final frame, the moment of film puncture.

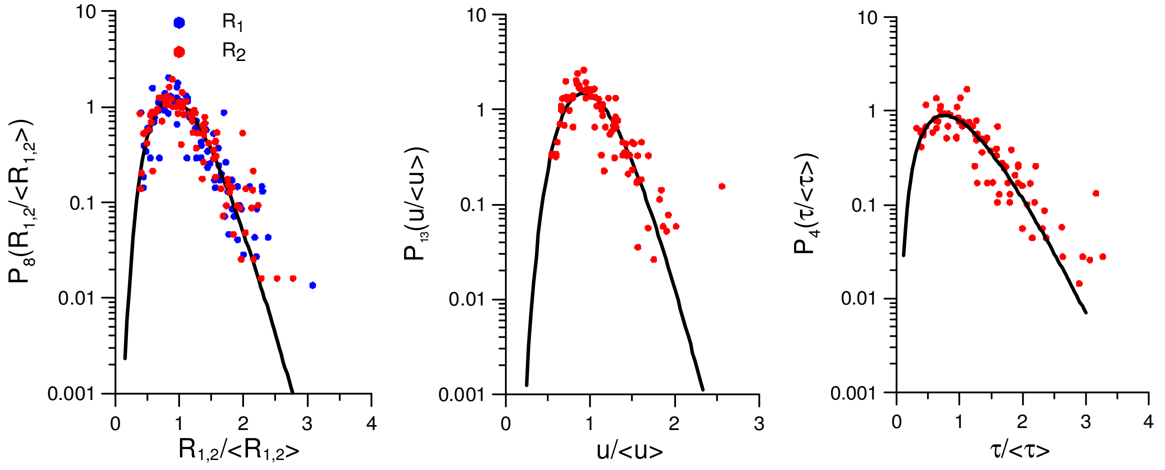

(A) (B) (C)

Fig. S3. (A) Distribution of "bags" over radii at the moment of nucleation ($R_1/\langle R_1\rangle$, blue symbols) and over radii at the moment of rupture ($R_2/\langle R_2\rangle$, red symbols); (B) Distribution of "bags" on velocities of edges ($u/\langle u\rangle$. (C) Distribution of "bags" on lifetimes ($\tau/\langle \tau\rangle$. The curves show the Gamma distribution for n = 8 (A) and n = 3 (B).

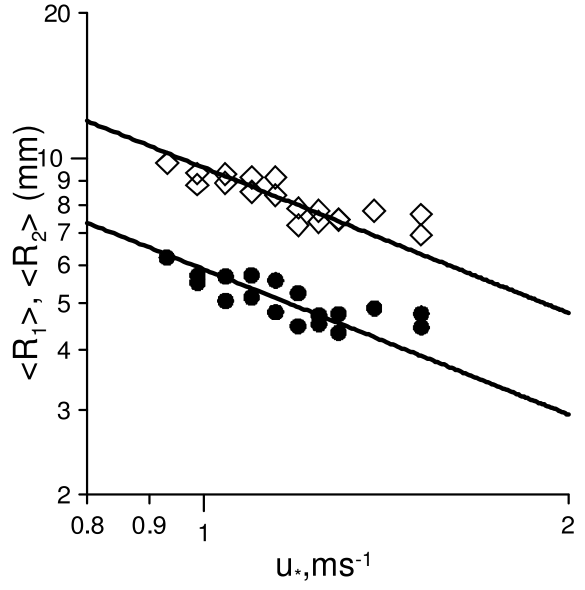

(A)

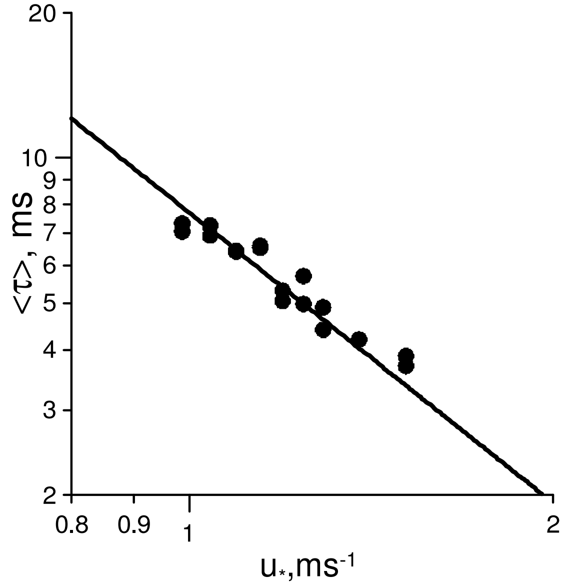

(B)

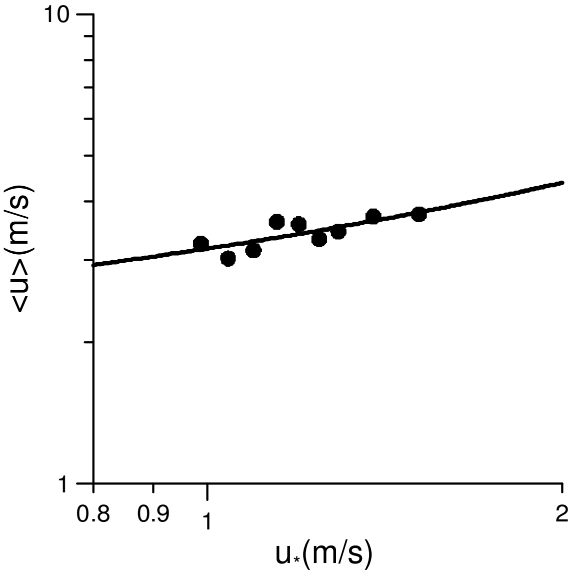

(C)

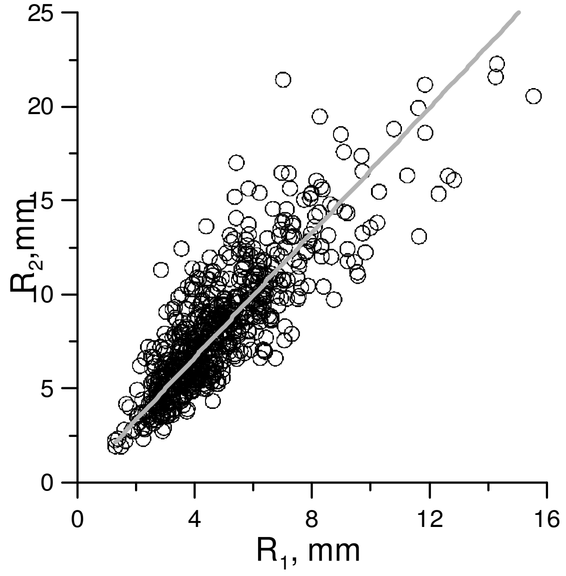

(D)

Fig. S4. (A)-(C) Dependencies on the friction velocity u_* of the following parameters of "bags": average initial $\langle R_1 \rangle$ (closed circles) and final $\langle R_2 \rangle$ radii (open diamonds), lifetime $\langle \tau \rangle$ and velocity of edges $\langle u \rangle$; (D) final radius of "bag" R_2 versus its initial radius R_1.

Movie S1

Breaking projection (top view)

Movie S2

Rupture of floating bubble (side view)

Movie S3

Formation and rupture of bag (side view)

Database S1

The database containing the data files in ASCII encoding:
n_events.dat - number of bags, projections and underwater bubble-bursting events via friction velocity,
P_size1.dat - distribution function of "bags" in the radius at the moment of nucleation ($R_1/\langle R_1 \rangle$,
P_size2.dat - distribution function of "bags" in the radius at the moment of rapture ($R_2/\langle R_2 \rangle$,
tol.dat - the distribution function of "bags" on their lifetime ($\tau/\langle \tau \rangle$,
size1_2_tol(u).dat - average initial and final radii of bags and their average life time via wind friction velocity,
f_.dat - initial R_1 and final $R2$ radii of "bags".
